# Moral Risk in the Behavior of Doctors of the Comprehensive Health Insurance in the Province of San Román, Puno-Peru, 2021

**DOI:** 10.3389/fpubh.2021.799708

**Published:** 2022-02-17

**Authors:** Julio C. Quispe Mamani, Yessica Quilca Soto, Dominga A. Calcina Álvarez, Cristóbal R. Yapuchura Saico, Nelly J. Ulloa Gallardo, Santotomas L. Aguilar Pinto, Betsy Quispe Quispe, Nelly B. Quispe Maquera, Balbina E. Cutipa Quilca

**Affiliations:** ^1^Faculty of Economic Engineering, National University of the Altiplano, Puno, Peru; ^2^Faculty of Health Sciences, Professional School of Dentistry, of the National University of the Altiplano, Puno, Peru; ^3^Faculty of Education of the Amazonian National University of Madre de Dios, Puno, Peru; ^4^Faculty of Engineering, Systems Engineering and Informatics of the National Amazonian University of Madre De Dios, Puno, Peru; ^5^Faculty of Administrative Sciences of the Andean University Néstor Cáceres Velásquez, Puno, Peru; ^6^Faculty of Accounting and Administrative Sciences, Professional School of Accounting Sciences, of the National University of the Altiplano, Puno, Peru

**Keywords:** physician behavior, moral hazard, health, health insurance, risk

## Abstract

**Objective:**

The objective of the research was to determine which socioeconomic factors are the ones that most influence the moral hazard in the behavior of the doctors of the Comprehensive Health Insurance in the province of San Román and to identify the attitude of the doctor to a gift and its influence in moral hazard.

**Methods:**

The methodology used has a mixed, non-experimental and correlational approach, the Binomial Probit econometric model was used, applying a survey to 32 active doctors who work in the different SIS centers.

**Results:**

It is concluded that the factors that influenced the moral hazard and the behavior of the doctors were the bad reputation with a positive relation (27%), the social pressure with a negative relation (98%) and the behavioral attitude with a positive relation (94 %).

**Conclusion:**

Of the survey carried out, 40.6% of doctors reject the offer of a gift or bribe, reducing the influence of moral hazard by 94%.

## Introduction

Moral hazard in health services is inevitable due to information asymmetry (1). In health insurance, the moral hazard of doctors is opportunistic behavior, that is, driven by their own economic interests, they take advantage of the information they have and thus increase the cost of medicines, in this way, ma -Maximizing your benefits and harming that of your patients (2–4).

Evans found that the doctor's moral hazard manifested itself in tests, drugs, and fees, such as unnecessary or expensive tests, and expensive or overused drugs (5).

Doctors benefit from a high degree of information asymmetry, which allows them to adjust their specific moral hazard behavior in different political settings, this has led to rapid growth in medical costs in several countries. Such is the case that health spending worldwide in 2019 grew very considerably, where on average it represents 10% of the gross domestic product (GDP) of the world economy; The World Health Organization (WHO) demonstrated the existence of an upward growth in health spending worldwide, where countries with low economic income increased their spending by an average of 6% annually, while countries with higher economic income made an average expense of 4% (6).

In the case of Latin America and the Caribbean, spending on health in 2017 averaged 1,000 USD / per person, where it only reached a quarter of the spending made by the countries that make up the OECD; Furthermore, in these countries it is necessary to develop improvements in the efficiency, effectiveness and good use of health spending, since by 2020, total health spending amounted to only 6.6% of GDP and was below 8, 8% highlighted by OECD countries. That is why, the democratic condition of (bio) ethics and (bio) politics implies that all its words, evaluations and moral judgments are not definitive, but contingent and reviewable (7–11). Not specifically in historical time, whose course modifies moral and political situations, but in the democratic motive, where value judgments can be expressed in an ethical, culturally diversified way, in legal form, in accordance with democratic laws, and in political form, according to the power interests of the various parties (12–14). There is not always an agreement, but they often enter into conflicts whose solution is in charge of the judicial criterion that, in turn, can be modified through the mobilization of ethical debate and electoral victory.

In addition, with the passage of time, the information economy focused on several fundamental aspects in contractual relationships, where it considers that one of the participants has an advantage over the other participant and only those situations in which the objectives are interesting of the participants are in conflict. Therefore, it is deduced from the strategic behaviors in the relationship between the agent and the principal, that the agent's objectives are in conflict with those of the principal. The amount paid is an income for the agent, but a cost for the principal; it is costly to the agent and the agent's effort benefits the principal. There is a moral hazard problem when asymmetric information occurs after the contract is signed (2).

The choice the choice of indebtedness is conditioned by the existence of information asymmetry between managers and shareholders, so that indebtedness is less when the utility of this decision is also less, that is, the asymmetry is less. At the same time, the capital structure is dependent on the importance of the information asymmetry with creditors, acting as a limit to its borrowing capacity (15). For example the probability of having a cesarean section controlling for the main risk factors of the population and for the hospital where it was performed is considerable, given that the choice of the hospital is endogenous (16).

In this sense, special attention should be paid to the mental health of health workers, especially women and nurses who are on the first line of treatment for patients with COVID-19. Generally speaking, when physicians and healthcare workers deal with the pain and suffering of other people, they are prone to stress, pain, depression, and compassion fatigue. Although the prevalence of these among health workers varies, they tend to increase; in the same way, in the face of this epidemic, it increases considerably. Lifestyle changes brought about by confinement and social distancing have generally placed a significant psychological burden on society (17).

Analyzing this behavior in Peru, it can be highlighted that the budgets assigned to the health sector by the governments in turn have not been sufficient (18); since the economic objective of the imposition of professional liability standards is to encourage diligent conduct by healthcare providers and to achieve a reduction in the number of medical errors or negligence, these are the purposes of these institutions. It is for this reason that the design of policies in the health sector that is in charge of the State, must consider the decisions and their behavior of the doctors who are subject to the establishment of the different incentives, since the neglect in this aspect generates the existence of moral hazard and adverse selection problems (19).

In 2019, the Universal Health Insurance (SIS) of the Department of Health (Minsa) had the largest affiliated population, reaching an average of 20, 200,000 insured, exceeding the 95% who reside in Peru that has coverage in the health service, being one of the regions with the most affiliates, Puno reached the number of 900, 000 affiliates. Regarding care and services, until October 2019 more than 19,373,000 external consultations, 522,000 hospitalizations and 348,000 surgeries were registered (20–22).

In 2020, the budget of the Health function increased by 23% compared to the previous year, leading to the amount of 6,514,285,714 USD; But even so, the health system continues to be exceeded, since said increase was to combat the problems generated by the pandemic and in compliance with Emergency Decree 017-2019, which produced new SIS affiliations (23).

From the aforementioned, by continuing to provide health services to users in a random, inconsistent manner and without reaching their expectations, it generates the probability of disease occurrence, where the group of individuals who are risk averse, will prefer Have health insurance in order not to be harmed in your income due to an unforeseen event in personal and family health. However, having insurance and health service distorts the choice of physicians when deciding when and how to provide the service, since they have a greater propensity to provide services, despite the fact that they are are not necessary, which generates a moral hazard for the doctor.

Therefore, evidencing the importance of this research, we sought to answer the following questions: What socioeconomic factors contribute to the existence of moral hazard in the behavior of comprehensive health insurance physicians in the province of San Román? and How does the doctor act when faced with a gift and does it influence the existence of moral hazard?

The objective of the research was to determine the socioeconomic factors that explain the moral hazard in the behavior of SIS doctors in the province of San Román-2021; in addition to identifying the doctor's attitude to a gift and its influence on moral hazard.

## Methods

The methodology used in this research has a mixed, non-experimental and correlational approach, using the bino-mial Probit econometric model, for which information was taken from the comprehensive health system. The sample size was determined by theoretical saturation; that is, a sampling was applied until a new sample no longer provides new information (24). In-depth interviews were carried out with 32 interviewees, using questions according to the objectives set out in this research, which was applied from May 2021 to June 2021 in the province of San Román (25).

### Methodology

The methodology used in this research has a mixed, non-experimental and correlational approach, using the binomial Probit econometric model, for which information was taken from the comprehensive health system. The sample size was determined by theoretical saturation; that is, a sampling was applied until a new sample no longer provides new information. In-depth interviews were conducted with 32 interviewees, using questions according to the objectives set out in this research, the same one that was applied from May 2021 to June 2021 in the province of San Román (26).

### Techniques

The techniques that were carried out is the selection of cases; development of a strict data collection protocol; data ordering; data analysis of the first case; theoretical sampling; reaching the close; and comparison of the newly constructed theory with existing theories and surveys.

### Population and Sample

#### Population

Reference was taken to active doctors who provide their services in the Comprehensive Health Insurance (SIS) of the province of San Román. Taking into account the 15 posts in which 1 or 2 average doctors work, and the Carlos Monge Medrano Hospital in which 10 average doctors work each year.

Having as population 35 doctors in the networks of San Román-Juliaca.

#### Sample

Active physicians who provide their services in the Comprehensive Health Insurance (SIS) of the province of San Román were taken as a reference. Taking into account the 15 posts in which 1 or 2 average doctors work, and the Carlos Monge Medrano Hospital in which 10 average doctors work each year. With a population of 35 doctors in the San Román-Juliaca networks.

#### Analysis of Variables

To find the factors that explain the moral hazard in the behavior of SIS doctors in the province of San Román-2021, the variables detailed in Table 1 were analyzed.

**Table 1:** Operationalization of model variables.

**Variable type**	**Variable**	**Notation**	**Concept**	**Categorization**
Dependent variable	Moral hazard in the behavior of doctors	RM	Does the respondent believe that there is moral hazard in the behavior of doctors?	Dichotomous 1 = Exists 0 = Does not exist
Independent variables	Salary	W	Average monthly salary of the doctor	Continuous and quantitative
	Work experience	EL	Years of work experience	Continuous and quantitative
	Bad reputation of the doctor	Rep	Does the respondent believe that the doctor's negative reputation influences moral hazard?	Dichotomous 1= Yes 0= No
	Social pressure toward doctors	PS	Does the respondent believe that social pressure influences committing moral hazard?	Dichotomous 1 = Yes 0 = No
	Medical ethics	EM	Does the respondent put into practice the principles of medical ethics?	Dichotomous 1 = Yes 0 = No
	Negative behavioral attitude of the doctor	AC	For the respondent, what would be their way of acting or behavioral attitude to a gift or bribe?	Dichotomous 1 = Positive 0 = Negative

#### Econometric Estimation of Moral Hazard in Physician Behavior

To determine the socioeconomic factors that explain the moral hazard in the behavior of SIS doctors in the province of San Román-2021, a binomial probit econometric model was applied, according to the following specification.


$$\begin{array}{l} Moral\;hazard\;in\;the\;behavior\;of\;doctors\\ =\beta_{0}{\;+\;\beta_{1}Salary+\beta }_{2}Work\;experience+\beta_{3}Bad\;reputation\;of\\ the\;doctor+\beta_{4}Social\;pressure\;towards\;doctors+\beta_{5}Medical\\ ethics+\beta_{6}Behavioral\;attitudes\;of\;the\;doctor+u_{t} \end{array}$$

## Results

To determine the factors that explain the moral hazard in the behavior of the SIS doctors in the province of San Román, the variables detailed in Table 1 were considered.

**Figure 1:**
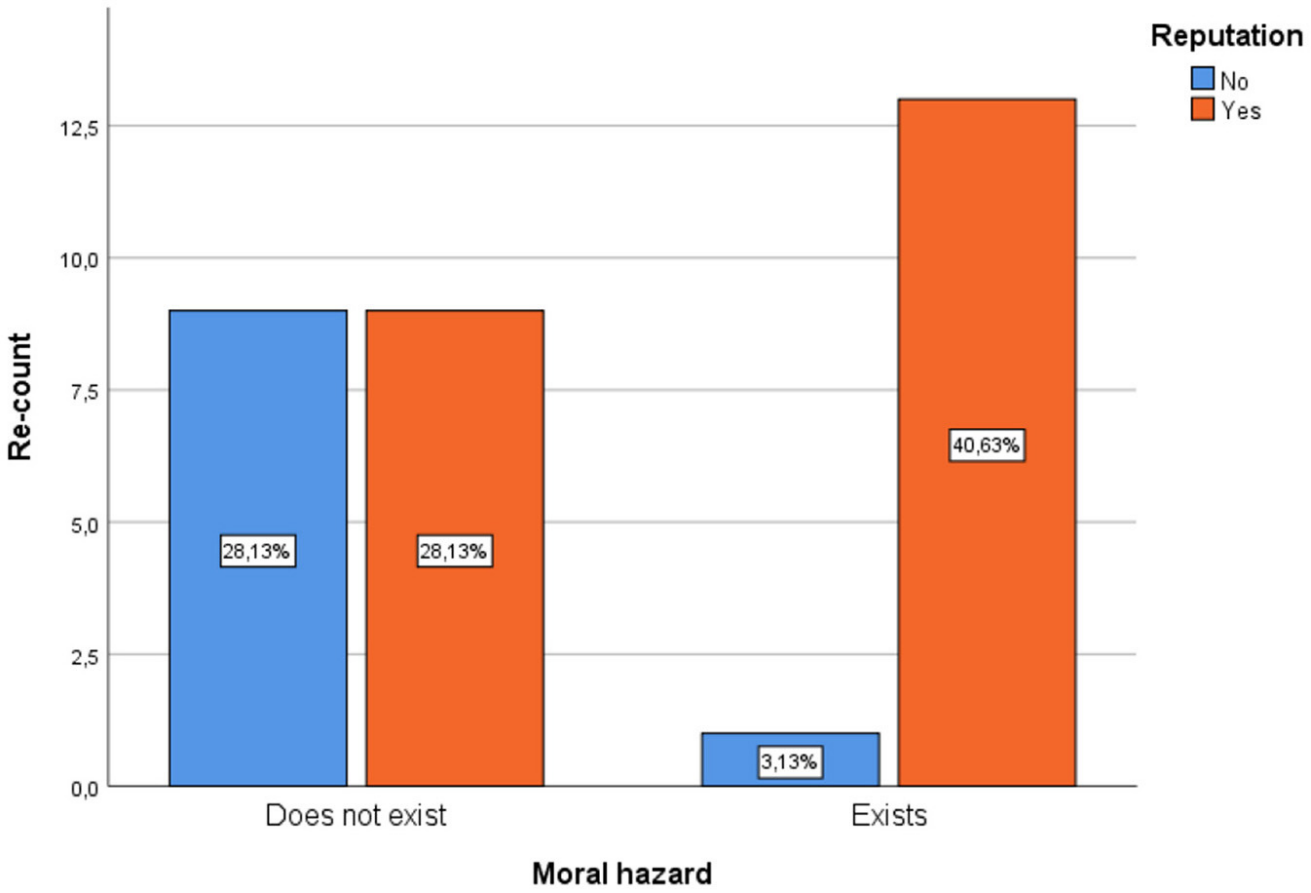
Relationship between moral hazard and bad reputation of the doctor.

Analyzing the relationship of moral hazard in the behavior of doctors with the bad reputation of the doctor, 40.63% of those surveyed consider that the bad reputation of doctors is one of the factors that influences the existence of moral risk in the behavior of Doctors, on the other hand, 28.13% consider that a bad reputation does not influence moral risk, and they consider that there is no moral risk in the behavior of doctors. What is shown is that the influence of this variable represents an important factor in the existence of moral hazard (Figure 1).

**Figure 2:**
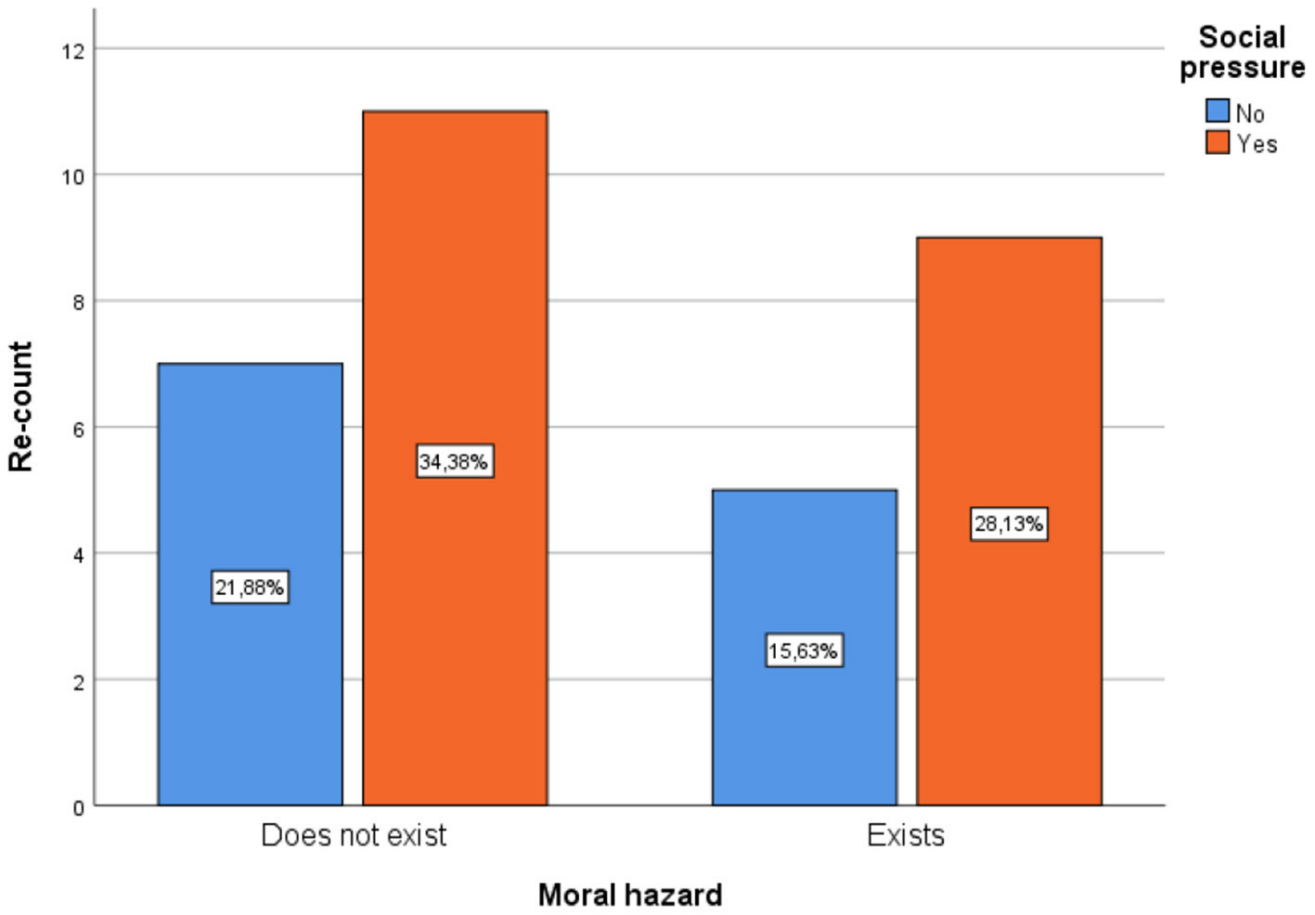
Relationship between moral hazard and social pressure.

Social pressure is the influence exerted by a social group to make a person change their attitudes, their thoughts or even their values. Therefore, of the total respondents, 34.4% indicate that social pressure does influence doctors to commit moral risk, but that they still think that there is no moral risk in the behavior of doctors, while 28.1%, being the second highest percentage, consider that social pressure does influence and lead the professional to risk. On the other hand, 21.9% indicate that it does not influence moral hazard and there is no moral hazard in physicians in general (Figure 2).

**Figure 3:**
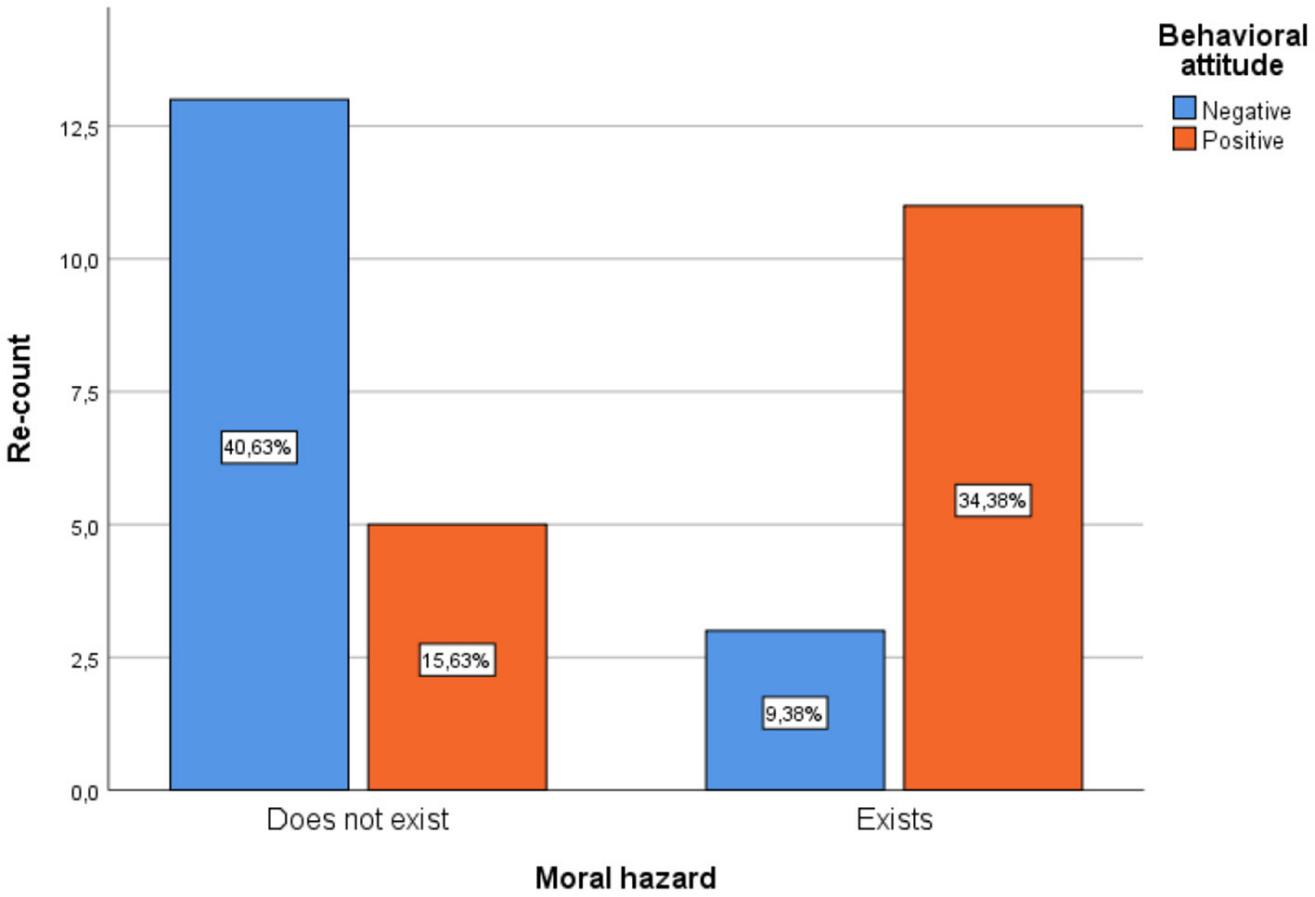
Relationship between moral hazard and negative behavioral attitude.

Another relevant variable is the negative behavioral attitude of the doctor, where 34.4% of the respondents stated that the behavioral attitude of acceptance in the face of a case of gift or bribery highly influences the moral risk of the doctors' behavior, while 15 6% considered that, although the attitude is of acceptance, it does not influence the moral risk and for them there is no moral risk. On the other hand, 40.6% indicated that the behavioral attitude of rejection in the face of a gift or bribe means that there is no moral risk, while 9.4% affirmed that, although their attitude is of rejection if there is moral risk, it which reflects that the behavioral attitude of the doctor does influence the moral risk of his behavior (Figure 3).

Analyzing the aforementioned, as long as the doctor has a negative reputation, the moral hazard is his behavior increases. Another important variable is the negative behavioral attitude of the doctor, which is positively related. Therefore, it could be said that while the behavioral attitude of the doctor is negative, the greater the moral risk in her behavior.

From all the aforementioned, applying the binomial probit econometric model, the results that are shown below were obtained, where the variables that are statistically significant to explain the moral hazard in the behavior of SIS physicians are: bad reputation of the doctor, social pressure toward doctors and negative behavioral attitude of the doctor, as observed in Table 2.

**Table 2:** Estimation of the binomial probit model.

**Probit regression**				**Number of obs**	**=**	**32**
				Wald chi2(6)	=	218.63
				Prob > chi2	=	0.0000
Log pseudolikelihood = −8.4899521				Pseudo R2	=	0.6129
**Moral hazard**	**Coef**.	**Std. Err**.	**z**	**P>z**	**[95% Conf. Interval]**	
Salary (W)	0.7717021	0.6205392	1.24	0.214	−0.4445325	1.987937
Work experience (EL)	0.3626199	0.4140363	0.88	0.381	−0.4488763	1.174116
Bad reputation of the doctor (Rep)	6.987361	1.045874	6.68	0.000	4.937485	9.037237
Social pressure toward doctors (PS)	−7.707817	1.568871	−4.91	0.000	−10.78275	−4.632887
Medical ethics (EM)	−2.191015	0.8429538	−2.60	0.009	−3.843174	−0.5388562
Negative behavioral attitude of the doctor (AC)	8.690894	1.631523	5.33	0.000	5.493167	11.88862
Constant	−7.996764	1.762656	−4.54	0.000	−11.45151	−4.542022

It can be seen in the regression presented that the goodness of fit indicator is 0.6129. This means that approximately 61.29% of the changes in the dependent variable “there is or there is no moral hazard” depend on the independent variables. Among the significant variables presented in the model are “bad reputation of the doctor,” which is positively related; and “the negative behavioral attitude of the doctor,” represented by the attitude that doctors give to a gift or bribe, keeping a positive relationship with the dependent variable.

Therefore, the greater the bad reputation of the doctor, the greater the probability of influence of moral hazard, in the same way, the greater the probability of negative behavioral attitude in a doctor, the greater the probability of in- moral hazard creep in the behavior of physicians.

The variable “Social pressure toward doctors” is a significant variable that is negatively related. That is, the probability that Social Pressure reduces moral hazard in the behavior of doctors.

In Table 3, the most relevant variables of the Probit model for each of the coefficients are explained as follows:

**Table 3:** Marginal effects.

**Variable**	**dy/dx**	**Std. Err**.	**z**	**P>z**	**[95% C.I.]**		**X**
Salary (W)	0.0059509	0.00591	1.01	0.314	−0.005628	0.017529	1.78125
Work experience (EL)	0.0027963	0.00378	0.74	0.460	−0.004614	0.010206	2.15625
Bad reputation of the doctor (Rep)	0.2657349	0.13222	2.01	0.044	−0.006597	0.524872	0.6875
Social pressure toward doctors (PS)	−0.9776822	0.03891	−25.13	0.000	−1.05395	−0.901415	0.625
Medical ethics (EM)	−0.0656182	0.05273	−1.24	0.213	−0.168966	0.03773	0.59375
Negative behavioral attitude of the doctor (AC)	0.9377471	0.07427	12.63	0.000	0.792174	1.08332	0.5

*Marginal effects after Probit y = Pr (RM) (predict) = 0.00248236*.

If the doctor's bad reputation increases, the probability of moral hazard in behavior increases by 26.57%.

With more social pressure toward doctors, the probability of moral hazard in behavior decreases by 97.77%.

The higher the negative behavioral attitude in physicians, the probability of moral hazard in behavior increases by 93.77%.

## Discussion

The doctor's current moral hazard behavior is divided into six categories: dereliction of duty, excessive surgery, excessive examinations and tests, excessive medication, excessive use of consumables, and excessive hospitalization (6).

From the analysis carried out, the results obtained coincide with who determined that accurate knowledge of the act of moral hazard of physicians and the trends of their change can be applied in many fields. In terms of hospital management, information asymmetry must be reduced and patient participation increased by redesigning the system and process. In addition, instruction in medical ethics should be improved so that physicians can declare the behavior of moral hazard and understand its consequences, avoiding it. In other words, particularly important when moral hazard affects the patient's health (6).

Just as they mention it, the explosive growth of medical schools could also be an indicator of the “perversion” of the system. While, on the one hand, it can be considered that such growth responds to the expectations of young people, who continue to opt for the medical career, it can also be thought that for some, such demands represent the opportunity to make a “good business” today, without examining the consequences of what will happen tomorrow, when the clinical field is lacking and when it becomes increasingly difficult to access a position in an oversaturated labor market (27).

In addition, we can consider that according to the findings of this research, most of the formal works are based on a retrospective remuneration system, which is the one that governs mostly in the American market and allows the increase in costs to be transferred to the fees. However, in most countries, healthcare providers do not have this option. Regulated formulas or public systems are identified precisely by having more prospective systems. In that case, the professional cannot increase his income by working longer nor can he generally select risks explicitly. Therefore, given a certain health system, and assuming that the selection of risks is not possible, variables such as reputation, risk signaling or altruism decisively influence the way of acting and constitute an excellent mechanism to enhance the quality or effort required of the supplier (19).

Also, propone to recreate a context of theoretical-empirical evidence in order to offer new contributions of argumentation and interpretation that allow to animate the political debate around the role of the financial responsibility of patients in medical services, at the national and subnational level. The decision to establish a strategy of co-responsibility between the insurer and the insured in health services: co-payment, moderating fee, among others. it is also a political issue that generates wide debate in relation to aspects of efficiency, equity and effectiveness of the health system, due to the problem of moral hazard that affects, to some extent, the level of quality proposed by the same policy (1).

We coincide with by determining that the relationship between the medical professional and his patients is the fundamental point in the activities of medicine. The so-called doctor-patient relationship, related to social changes, to the development of the individual and society, the notions of value and personal respect, autonomy of the person and the recognition of the fundamental rights of people. The idea of respect for the rights of patients and the voluntary consent of the patient in their treatment, modified the action of doctors in the process of health care and made them a more egalitarian relationship between the doctor and the patient. The RMP was traditionally strongly dominated by the expert in medicine and the patient who requires their services, they always had an inferior position, this relationship called medical paternalism prevented the patient from making use of their rights and decision-making capacity, while leaving all responsibility for the care of the disease to the doctor, which at present has ceased to be acceptable and has come to an end (28).

Furthermore, it is argues that information asymmetries can be very crucial in insurance markets and can also lead to very strong inefficiencies. Therefore, when measuring data asymmetries and finding a positive relationship between the selected coverage and the insured's risks, it is important to clearly identify the causal relationship behind this relationship. If moral hazard behaviors only generate an increase in premiums, on the contrary, adverse selection can lead to more serious market failures and stabilize the proper functioning of insurance markets (29).

Complementarily, the insurance and affiliation to some regime increases the probability of consulting health care services for prevention reasons (presence of ex ante moral hazard). In addition, when you have complementary health programs, the probability of consulting for prevention increases (people do not stop preventing diseases by the fact of acquiring medical insurance). The presence of ex post moral hazard in members of the health system was not clearly found; however, when you have the coverage of the SGSSS (contributory regime, for example) if it generates it and directly; that is, it has a greater preference for medical consultation compared to the alternative of having complementary programs, which indicates that insurance increases the probability of choosing to go to the doctor or health services institution. A sign of the presence of ex post moral hazard is related to the value to be paid for health care services: the lower the value to be paid, the greater the probability of going to the medical institution of medical services (30).

Finally, we agree when considering that 50% of the population has difficulty adhering to treatment, which can be observed through the improvement of patients and the objective of estimating the prevalence of drug compliance problems. Improved patient perception is the most important factor predicting medication adherence. Information asymmetry, also known as “information failure,” occurs when one party to an economic transaction has more material knowledge than the other. For example, doctors generally understand medical practice better than patients. One of the consequences of information asymmetry is adverse selection, which describes a phenomenon in which an insurance company discovers the possibility of extreme losses due to undisclosed risks when selling policies (31).

## Conclusions

The most relevant socioeconomic factors that affected the moral hazard and behavior of the doctors of the Integral Health System in the province of San Román were: the bad reputation of the doctors, the social pressure and the negative behavioral attitude in the doctors. If the doctor's bad reputation increases, the probability of moral hazard in behavior is 26.57% with positive relationship. The more social pressure on doctors, the probability of moral hazard in behavior is 97.77% with a negative relationship. The greater the negative behavioral attitude in physicians, the probability of moral hazard in behavior is 93.77% with positive relationship.

40.6% of doctors indicate that rejecting the offer of gift or bribery causes cases of moral hazard to decrease, this due to their positive relationship of 94%, which indicates that if the number of cases of acceptance of gifts or bribes increases (or decreases), the existence of moral hazard increases (or decreases) by 94%.

Finally, it is necessary to continue developing research of this type oriented at the macro level, perhaps also applying it to workers in other productive sectors such as education, transportation, local governments, regional governments and sectoral governments that are part of the government in turn. Complementarily, it is necessary to evaluate the analysis of the moral hazard that may exist in the personnel working in the private sector, an issue that has not been widely studied to date.

## Data Availability Statement

The original contributions presented in the study are included in the article/supplementary material, further inquiries can be directed to the corresponding author.

## Author Contributions

All the authors listed have made a substantial and important contribution to the development of this scientific article, directly and intellectually, and approved their full publication of the document.

## Conflict of Interest

The authors declare that the research was conducted in the absence of any commercial or financial relationships that could be construed as a potential conflict of interest.

## Publisher's Note

All claims expressed in this article are solely those of the authors and do not necessarily represent those of their affiliated organizations, or those of the publisher, the editors and the reviewers. Any product that may be evaluated in this article, or claim that may be made by its manufacturer, is not guaranteed or endorsed by the publisher.
